# Gliadin Sequestration as a Novel Therapy for Celiac Disease: A Prospective Application for Polyphenols

**DOI:** 10.3390/ijms22020595

**Published:** 2021-01-08

**Authors:** Charlene B. Van Buiten, Ryan J. Elias

**Affiliations:** 1 Department of Food Science and Human Nutrition, College of Health and Human Sciences, Colorado State University, Fort Collins, CO 80524, USA; 2 Department of Food Science, College of Agricultural Sciences, Pennsylvania State University, University Park, PA 16802, USA; elias@psu.edu

**Keywords:** celiac disease, polyphenols, epigallocatechin gallate, gluten, gliadin, protein sequestration

## Abstract

Celiac disease is an autoimmune disorder characterized by a heightened immune response to gluten proteins in the diet, leading to gastrointestinal symptoms and mucosal damage localized to the small intestine. Despite its prevalence, the only treatment currently available for celiac disease is complete avoidance of gluten proteins in the diet. Ongoing clinical trials have focused on targeting the immune response or gluten proteins through methods such as immunosuppression, enhanced protein degradation and protein sequestration. Recent studies suggest that polyphenols may elicit protective effects within the celiac disease milieu by disrupting the enzymatic hydrolysis of gluten proteins, sequestering gluten proteins from recognition by critical receptors in pathogenesis and exerting anti-inflammatory effects on the system as a whole. This review highlights mechanisms by which polyphenols can protect against celiac disease, takes a critical look at recent works and outlines future applications for this potential treatment method.

## 1. Introduction

Gluten, a protein found in wheat, barley and rye, is the antigenic trigger for celiac disease, an autoimmune enteropathy localized in the small intestine. Initial symptoms of celiac disease are gastrointestinal discomfort as well as increased permeability and inflammation of the small bowel. Long-term exposure to gluten can cause extensive damage of the small intestine, leading to impaired nutrient absorption and chronic conditions related to malnutrition [1]. Celiac disease is one of the most prevalent autoimmune diseases in the world, affecting approximately 1% of the population in both Europe and the United States [2]. Understanding of the magnitude of this disorder has continued to grow over the last decade as prevalence has been revealed, thanks to large epidemiological studies and improved serologic tests employing the use of the most up-to-date methods of disease identification [3]. Interest in celiac disease from a public health perspective has mirrored this increase in prevalence, increasing five-fold over the last 30 years [4].

Despite heightened public awareness and understanding of celiac disease, the only reliable treatment strategy is eliminating gluten from the diet altogether, which can be both inconvenient and a financial burden [5]. A variety of alternative strategies have been explored, from gut barrier-modifying pharmaceuticals and biological immunosuppressants [6,7] to treatments targeting the antigen itself rather than the physiology of the patient. The pathogenesis of celiac disease depends largely on the physical features of gluten proteins and structural recognition of digestion-derived immunostimulatory epitopes found in the gliadin subunits of gluten proteins [8], making direct interactions with gluten protein a therapeutic option in the treatment of celiac disease. Recent studies suggest that preventing the digestion and absorption of gluten proteins by sequestering the protein from interaction with the gastrointestinal tract may be an effective, novel therapy for celiac disease. This has been successfully demonstrated in vivo with the administration of a synthetic polymer which forms stable complexes with gluten proteins [9,10]. However, natural and nutraceutical options for this application have also been explored in vitro, including sequestration by dietary polyphenols [11,12,13].

Polyphenols are a structurally diverse class of secondary plant metabolites that have been shown to have both beneficial and detrimental effects on human health. As potent antioxidant and anti-inflammatory agents, polyphenols have been investigated as natural therapeutics for chronic inflammatory diseases such as allergies and inflammatory bowel disease (IBD); however, they also can elicit anti-nutritional effects upon consumption by humans and animals alike due to their ability to inhibit digestive enzymes and interact with other dietary compounds, including proteins [14,15]. Interactions between polyphenols and proteins have been explored as potential therapeutic strategies for food allergies, showing that immunoglobulin (Ig) E responses can be reduced and digestion of allergenic proteins can be blocked [16,17,18,19]. Gliadins, a subunit of gluten, make for a particularly interesting protein target for polyphenol interactions as they are rich in proline residues and possess natively unfolded structures with polyproline II helical (PPII) motifs; these features have been shown to favor interactions with polyphenols [20,21]. These characteristics, in addition to their widespread intake in the human diet, make polyphenols an attractive option for therapeutic research and development within the context of celiac disease.

Recent studies suggest that the anti-nutritional characteristics of polyphenols including flavonoids and procyanidins can be used to physically sequester gliadin proteins, disrupting key steps in disease pathogenesis in vitro. This potential treatment strategy features a variety of advantages including ease of incorporation into the diet, developed understanding of toxicity risks and a large body of work supporting supplementation of these compounds to treat other ailments. The objective of this review is to discuss gliadin sequestration via polyphenol interaction as a therapeutic strategy for managing celiac disease as well as potential mechanisms and future applications of polyphenols as bioactive therapeutic agents.

## 2. Gluten Proteins

Gluten is a protein found primarily in grains of the *Triticeae* tribe of *Poaceae* cereal grasses including food grains such as wheat, barley and rye [22]. It is heterogeneously composed of nearly 40 highly homologous proteins classified by two subunits—glutenins, a cysteine-rich class of fibrous proteins, and gliadins, which are classified as prolamins due to their richness in proline and glutamine residues [23].

Prolamins are storage proteins which are soluble in organic solvents and are found in a variety of cereal seeds. They make up approximately half of the total protein found in mature grains, with the exception of oats and rice [22,24]. While all prolamins are rich in proline and glutamine, they are structurally diverse, ranging from 10 to 100 kDa [23]. Further classification of prolamins differentiates proteins by their molecular weight (MW) and relative sulfur (S) content, creating the groupings high-MW prolamins (6–10% total prolamins), S-rich prolamins (70–80%) and S-poor prolamins (10–20%) [22].

The sulfurous component of prolamins is cysteine. Cysteine frequency in the S-rich class of prolamins is 2–3% compared to 30–40% glutamine and 15–20% proline [22], but it is cysteine’s ability to form disulfide bonds that is of key importance with respect to the functionality of gluten during the production and processing of grain-based food products. The viscoelasticity of gluten protein is a product of inter-and intramolecular disulfide bond formation between glutenins and gliadins, respectively, forming a gluten protein network, which contributes to the functional characteristics of gluten within a food product [25,26].

Gliadins, rather than glutenins, have been implicated as being responsible for the onset of the inflammatory and immune responses in celiac disease pathogenesis, though repeat motifs conserved in both subunits of gluten are recognized as immunostimulatory epitopes [27]. In particular, short repetitive sequences of multiple glutamine residues and adjacent proline residues have been noted for heightened immunotoxicity alone or when embedded within longer gliadin peptides [21,28].

## 3. Celiac Disease Pathogenesis

Celiac disease manifests in the small intestine, which is the site of gluten protein digestion and absorption in the gastrointestinal tract. As luminal and brush border enzymes are secreted, digestive enzymes hydrolyze gluten proteins into free amino acids and di- and tripeptides by targeting specific cleavage sites dependent on the enzyme [29]. Digestive enzymes produced by the stomach, pancreas and brush border lack the ability to fully hydrolyze proteins with high frequencies of proline residues, which is a characteristic of gluten proteins. This makes gluten, particularly gliadins, exceptionally resistant to enzymatic hydrolysis during digestion in all individuals, regardless of whether they suffer from celiac disease or not [30]. These undigested fragments of gliadin cross the brush border via transcellular and paracellular mechanisms [31,32,33], allowing them to stimulate a host of deleterious effects on the small intestine including cytotoxicity, immunomodulation and gut permeation [34].

One mechanism of gliadin transport across the brush border is protected transcytosis due to abnormal expression of CD71 in the enterocytes of individuals with celiac disease. CD71 has been shown to protect gliadin from lysosomal degradation during transcytosis upon complexation with IgA, blocking the further degradation of immunogenic peptide fragments and allowing them to enter the lamina propria intact. This has been shown to occur in active cases of celiac disease where patients demonstrate elevated luminal anti-gliadin IgA as a response to exposure, as opposed to individuals adhering to a gluten-free diet [35]. Paracellular leakage of gliadin peptides into the lamina propria has also been explored [32,34,36,37]. Studies have shown that gliadin digestive products are able to bind to the luminal chemokine receptor CXCR3, the expression of which is elevated in active celiac disease. Binding to this receptor stimulates the recruitment of myeloid differentiation factor 88 (MyD88). MyD88 induces the release of pro-inflammatory cytokines including interleukin (IL)-15, and zonulin, a protein archetype which mediates intestinal barrier function [32].

Though dysregulated expression of IL-15 has been implicated in many inflammatory autoimmune diseases [38], it is considered to be the hallmark of celiac disease due to chronic upregulation in the intestinal epithelium and lamina propria [39]. The primary role of IL-15 in celiac disease pathogenesis is to signal for lymphocytes to infiltrate the submucosa [39,40]. Overexpression of IL-15 interferes with normal immune homeostasis by preventing transforming growth factor (TGF)-β from suppressing T cell activation [41]. As a result, the intestinal mucosa of individuals with active celiac disease features abnormally high levels of intraepithelial lymphocytes (IELs). These IELs contribute to villous atrophy and overall mucosal damage observed in active celiac disease by killing intestinal epithelial cells (IECs) producing stress signals, including IL-15 [39].

Zonulin proteins, which are MyD88 dependent and produced by IECs [42], can trigger the disassembly and downregulation of intercellular junction proteins between IECs by directly activating the epidermal growth factor receptor or indirectly via proteinase-activated receptor 2, effectively mediating the permeability of the intestinal barrier [34]. The intercellular junction proteins of note in these pathways are tight junction (TJ) and adherent junction (AJ) proteins, which are found in polarized epithelial cells such as those in the intestinal mucosa. They serve as molecular gates to the body, regulating the transport of nutrients and protecting the body from antigens, toxins and macromolecules [43]. TJs and AJs are dynamic structures within the mucosa, able to be modified by a variety of stimuli including nutrients, cytokines, toxins and pathogenic bacteria [44].

Disassembly of TJs and AJs occurs via vesicular transport of proteins from the cell membrane to alternative locations within the cell, or by rearrangement of the actin cytoskeleton. Gliadin-stimulated zonulin release has been shown to modify junctional properties of IEC lines IEC-6 and Caco-2, altering the localization of TJ proteins claudin-3 and -4 and causing the polymerization of actin filament [45,46]. Exposure of gliadin also downregulates the expression of some TJ and AJ proteins, including E-cadherin, occludin and zonula occludins-1 (ZO-1) [45]. These alterations to junctional proteins decrease the functionality of the intestinal barrier, increasing permeability and allowing leakage of small molecules into the lamina propria. This increase in paracellular flux provides an alternative route for gliadin to cross the brush border [34].

The adaptive immune response associated with celiac disease is largely based on the interaction between gliadin peptides and an endogenous enzyme called transglutaminase 2 (TG2). Early studies implicating TG2 in celiac disease pathogenesis showed that jejunal tissue samples taken from individuals with treated and untreated celiac disease demonstrated increased TG2 activity compared to healthy controls [47]. While the prevailing hypothesis has been that TG2 activity occurs in the lamina propria [48], recent studies have shown that active TG2 can also be released into the lumen as a result of standard shedding of the gut epithelium [49], where anti-TG2 B cells may develop to produce autoantibodies against TG2 [49,50]. Catalytically active TG2 plays an important role in the presentation of gliadin as an antigen; it catalyzes the deamidation of glutamine to glutamic acid within gliadin peptides [48], resulting in a change in charge of the peptide from neutral to negative [48,51]. This negative charge increases the affinity for gluten peptides to form major histocompatibility complex (MHC) class II complexes with HLA-DQ2 and HLA-DQ8 molecules on antigen presenting cells (APCs) [52]. Furthermore, when TG2 binds to gliadin for deamidation, it forms a transient gliadin–TG2 complex. This complex can be recognized as an antigen by HLA-DQ2 and HLA-DQ8 MHC class II molecules in a similar fashion to the recognition of the peptide alone. However, in this case, TG2 is also recognized and presented as an antigen despite its endogenous production by epithelial cells.

The adaptive immune response associated with celiac disease is initiated by MHC class II molecules on the surface of APCs in the lamina propria. These MHC class II molecules are able to bind to deamidated gliadin as well as gliadin–TG2 complexes, presenting each as antigens to naïve T cells. MHC class II molecules have been found to preferentially bind to the main chain of peptide ligands through hydrogen bonding [53,54]. Presentation of gliadin and gliadin–TG2 by MHC class II molecules activates T helper (Th) cells in the lamina propria [55]. Upon recognition of gliadin and TG2, gliadin-reactive CD4^+^ cells follow the Th1/Th0 pathways and release anti-inflammatory cytokine IL-10 and pro-inflammatory cytokine interferon (IFN)-γ, which activates signal transducer and activator of transcription 1 (STAT1) and interferon regulatory factor 1 (IRF1) [56]. IL-10 is upregulated in active celiac disease [57], and IFN-γ is responsible in part for mucosal damage by stimulating the release and activation of matrix metalloproteinases [40,58]. Mucosal damage is also carried out by CD8^+^ cytotoxic T cells, which are stimulated by the release of cytokines from the CD4^+^ T cells [40]. CD4^+^ T cells further propagate the immune response by activating effector B cells, or plasma cells, which produce anti-gliadin and anti-TG2 IgA and IgG antibodies. In contrast to most other instances of T cell-dependent activation of B cells, B cell activation in celiac disease does not result in the production of memory B cells. As a result, gliadin and TG2 antibodies disappear from circulation after approximately one month of following a gluten-free diet [59].

Presentation of symptoms of celiac disease function on a gradient of exposure. For some individuals with celiac disease, as little as 50 mg of gluten per day is enough to elicit a physiological response [60] in the blanket form of “gastrointestinal discomfort”, including diarrhea, abdominal pain, bloating, and constipation [61]. Other physical symptoms result from long-term gluten exposure. This occurs often when individuals with celiac disease are asymptomatic, but present illnesses related to nutrient absorption such as iron deficiency and reduced bone mineral density [62].

The most telling diagnostic feature of celiac disease is the degradation of small intestinal mucosal architecture including surface damage to enterocytes, infiltration of IELs and the blunting of villous structure, which results in the loss of small intestinal surface area [63]. As celiac disease is characterized by a heightened immune response, celiac disease patients often also demonstrate a greater number of IELs in the mucosa compared to healthy controls [64]. The severity of intestinal damage is measured by changes in the mucosal architecture of the small intestine—namely, crypt hyperplasia and villous atrophy. In a healthy small intestine, the surface area of the organ is increased by numerous folds and villi, which are vascularized projections on the surface of the folds that are lined by epithelial cells, creating the brush border of the small intestine and increasing surface area for efficient nutrient absorption. Intestinal crypts are indentations within the small intestine that comprise stem cells and are responsible for the renewal of brush border epithelial cells during normal epithelial shedding [65]. In celiac disease, crypts can become hyperplastic due to increased proliferation of the cells they contain. In this case, the crypts become shallow and eventually disappear completely, leading to a flattening of the small intestinal surface [66]. Similarly, the villi become atrophic as a result of the onslaught of inflammation and cytotoxicity stimulated by gluten ingestion, shortening at first and eventually wearing down to the point of the intestinal surface becoming completely flat [64].

## 4. Current Treatment Strategies Undergoing Clinical Trial

As of 2021, the only reliable method for avoiding the symptoms and intestinal damage associated with celiac disease is adherence to a gluten-free diet. Despite the inconvenience and financial cost of a gluten-free diet [5], it has been proven in case-controlled studies to be an effective strategy for the elimination of gastrointestinal symptoms. Gluten was identified as the dietary component of interest in celiac disease in 1953, a realization that was closely followed by dietary intervention studies confirming the findings [67,68,69]. In an American study of 215 patients with celiac disease, 76% of participants reported the subsiding of abdominal pain and 41% reported decreased frequency of diarrhea after 6 months of a gluten-free diet. In a majority of these cases, symptoms subsided in less than one month on the gluten-free diet [70]. Further, a gluten-free diet can reverse mucosal damage. In a prospective study of 65 Italian adults, 66% of the participants achieved full histological recovery after one year of adhering to the gluten-free diet and another 32% achieved partial recovery [71].

The downsides of the gluten-free diet include increased food costs and nutritional implications [5]. One study tracking the diets of adolescents following a gluten-free diet showed macronutrient imbalance by excessive protein and fat consumption and low amounts of carbohydrates. Patients were also lacking in intake of fiber and, interestingly, calcium and iron [72]. Based on this finding, it appears that following a gluten-free diet may not help resolve some of the absorption-related nutritional deficiencies that patients present at the time of diagnosis.

Many strategies have been explored to help individuals with celiac disease maintain normal diets without the restrictive nature of the gluten-free diet. These strategies have come in the form of both synthetic and naturally-derived options, and target multiple stages of celiac disease pathogenesis by interacting directly with either the body or with gluten proteins. Table 1 lists the completed and ongoing clinical trials listed by ClinicalTrials.gov for novel celiac disease treatments beyond eliminating gluten from the diet. Of the 58 trials summarized, 37 have been designated as completed, two have been terminated, one has been suspended and the remainder are in various stages of recruitment.

### 4.1. Pathophysiology-Targeted Therapies

Celiac disease pathophysiology is based on an overreactive immune response upon exposure to gluten proteins. The sensitization of the digestive system to gluten, initiation of intestinal permeability and upregulation of immune signaling observed in celiac disease are all therapeutic targets which have been explored in clinical trials over the last two decades. Table 1 outlines 38 clinical trials targeting the pathophysiology of celiac disease, 35 of which have been completed with one trial being terminated. 

#### 4.1.1. Sensitization

Gluten sensitization and tolerance development has been explored with Nexvax2, a vaccine comprising peptides recognized by CD4^+^ T cells in individuals with the HLA-DQ2 haplotype that is administered subcutaneously on a weekly basis [73]. While Phase 1b clinical trials did not show complete desensitization, improvement of the celiac disease response was observed as decreased T cell mobilization [74] and decreased IL-2 production in response to injected gluten peptides [75]. However, a review of histological data from Phase 1 and 2 trials suggests that Nexvax2 is not as effective in preventing the formation of mucosal lesions as seen by duodenal biopsies [76], and its lack of efficacy in Phase 2 trials in comparison to a placebo resulted in the discontinuation of the trials in June 2019 [77].

#### 4.1.2. Gut Barrier Function Enhancement

The disruption of the gut barrier is a primary step in celiac disease pathogenesis. Enhancement of barrier function and prevention of gut permeability have both been explored as potential therapeutic options for celiac disease.

Probiotics have also been shown to improve barrier function in both in vitro and in vivo models of celiac disease. *Bifidobacterium longum* CECT 7347 and *Lactobacillus casei* ATCC 9595 have been shown to improve gut barrier function, reduce inflammation and repair gliadin-mediated intestinal damage in gluten-sensitized mice [78,79], and Bifidobacterium lactis has demonstrated protective effects against gliadin-mediated IEC permeability and TJ disruption in vitro, observed by immunofluorescent microscopy. It has been hypothesized that this preservation of barrier integrity is due to the upregulation of cyclooxygenase (COX)-1 while concurrently downregulating COX-2, a balance that favors the mucosal membrane maintenance while preventing inflammation [80].

As perhaps the most promising pharmaceutical option for treating celiac disease, larazotide acetate also targets barrier function as a protective mechanism. Larazotide acetate is a synthetic 8-amino acid peptide that preserves gut barrier function by acting as a zonulin antagonist and preventing the disruption of tight junction proteins stimulated by gliadin. In vitro, larazotide acetate has been shown to prevent gliadin-mediated permeability in Caco-2 cells by preserving tight junction proteins, an effect which also prevented the paracellular transport of FITC-labeled gliadin [81]. Based on the idea that the paracellular transport of gliadin is a rate-limiting factor in pathogenesis, larazotide acetate has been shown to effectively prevent the destruction of IECs and the associated immune response that occurs when gliadin is able to transcend the intestinal barrier. This has been demonstrated in both transgenic HLA-HCD2/DQ8 mice [82] and in clinical trials. Clinical trials for larazotide acetate have shown remarkable success in safety and tolerability as well as improvement of intestinal permeability and histopathology [83,84]. As of June 2018, larazotide acetate has advanced to Phase 3 clinical trials. In these trials, the efficacy of the drug will be measured based on celiac disease patient-reported outcomes (CeD PRO) of abdominal domain scoring, including the frequency of abdominal cramping, abdominal pain, bloating and gas (NCT03569007).

#### 4.1.3. Immunosuppression

Suppression of the immune system has been explored via the administration of both natural and synthetic agents. Natural immunosuppressants including probiotics such as *Bifidobacterium bifidum* IATA-ES2 and *Bifidobacterium longum* ATCC 5707, which have been demonstrated to suppress IFN-γ secretion by peripheral blood mononuclear cells (PBMCs) in the presence of gliadin in vitro [85,86]. Data from clinical trials supporting the use of probiotics to treat celiac disease in humans have shown varied results, especially concerning the impact on histological outcomes. However, a clinical trial using *Bifidobacterium infantis* showed reduced serologic markers after three weeks of supplementation alongside a gluten-containing diet compared to the controls, and patients noted an improvement of gastrointestinal symptoms [87]. Natural immunosuppression has also been achieved by intestinal colonization with helminths. *Necator americanus*, or hookworms, have been shown to reduce gluten-mediated IFN-γ prevent mucosal damage in celiac disease patients. In a small clinical trial of 12 adults with celiac disease, infection with *N. americanus* preserved intestinal histopathology upon gluten challenge as measured by Marsh scores and IEL counts per 100 enterocytes [88].

Immunosuppression has also been achieved in pre-clinical and clinical trials exploring direct antagonists to a mounted immune response. Injection with anti-IL-15 antibody TM-β1 has been shown to reverse abnormally high CD8^+^ T cells in transgenic mice as well as improve villous architecture and reduce mucosal lesions [89]. Similarly, vedolizumab, an a4β7 integrin antibody, has been successful in disrupting the trafficking of IELs in the gut in Phase 2 clinical trials of individuals with IBD, leading to remission in 33% of patients receiving intravenous treatment versus 14% receiving the placebo [90,91]. Though Phase 2 trials investigating vedolizumab in celiac disease patients have been terminated per ClinicalTrials.gov, etiological similarities between IBD and celiac disease suggest that this may still be an effective treatment option to explore in the future. Immunosuppression has also been achieved by an orally administered chemokine receptor (CCR) 9 antagonist called Vercirnon, formerly known as CCX282-B. CCR9 is a receptor expressed on CD4^+^ and CD8^+^ T cells that is involved with the recruitment and homing of those cells to the small intestine, which ultimately results in the destruction of the small intestinal mucosa during celiac disease [92]. The efficacy of Vercirnon has been demonstrated in Phase 2 clinical trials for Crohn’s disease, which demonstrates mucosal T cell migration similar to that observed in celiac disease. Daily oral administration of 500 mg vercirnon resulted in decreased mean Crohn’s Disease Activity Index scores in 61% of patients after 12 weeks compared to a 47% response rate in the placebo group [93].

#### 4.1.4. Disruption of Antigen Presentation

Once gliadins have passed the gut barrier and an inflammatory response is mounted, celiac disease pathogenesis proceeds by an adaptive immune response where gliadins, alone or bound to TG2, are presented as antigens. Inhibiting or otherwise impairing antigen presentation has been shown to be an effective strategy through several approaches. One approach focuses on impairing the processing of gliadin peptides required for antigen presentation. As previously discussed, deamidation of glutamine residues by TG2 greatly enhances the affinity of the peptide for the MHC class II binding pocket. One study found that incubation of gliadins with synthetic “blocking peptides” reduced TG2 activity in vitro by up to 36% [94]. Synthesis of gluten epitopes with azidoproline in the place of two proline residues demonstrated the efficacy of a similar “blocking” approach at the antigen presentation level, as the modified epitopes were able to bind to HLA-DQ2 binding pockets and prevent T cell proliferation, though the binding was not competitive enough with unmodified gliadin to pursue in vivo testing [95]. Binding optimization experiments have led to the development of synthetic peptides that are able to bind to HLA-DQ2 with 100–200-fold greater affinity than gliadin epitopes and modified versions of an immunodominant gliadin 33-mer, though they have demonstrated varying success in their ability to prevent T cell activation [96,97].

Direct inhibition of TG2 has been explored ex vivo with active-site TG2 inhibitor R283. Pre-treatment of tissue with R283 prevented gliadin-mediated T cell activation in duodenal biopsies, but the effect was diminished when gliadin had been deamidated prior to addition to the culture, suggesting that the prevention of deamidation was the critical factor at play [98]. A similar approach has been taken with the pharmaceutical therapy Zedira, or ZED1227, which irreversibly blocks TG2. In a mouse model of intestinal inflammation, ZED1227 has been shown to reduce TG2 activity between 2- and 4-fold after intraperitoneal injection [99]. This therapy has been investigated for safety and tolerability in Phase 1 clinical trials and has recently advanced to Phase 2 trials for efficacy in celiac disease treatment [100,101].

### 4.2. Gliadin-Focused Therapies

While many of the pathogenesis-focused therapies target events after passage of gliadin to the lamina propria, gliadin-focused therapies instead modify the immunological potential of gluten. Methods for detoxification of gliadin proteins include enzymatic supplementation to further hydrolyze immunostimulatory proteins or prevention of breakdown and recognition altogether through protein sequestration. Table 1 outlines 20 clinical trials for gliadin-focused therapies, 12 of which have been completed with one terminated and one suspended for administrative reasons.

#### 4.2.1. Enzymatic Hydrolysis

The incomplete digestion of gliadin in the lumen observed in individuals with celiac disease has been explored as a therapeutic target with varying levels of success. Despite the proteolytic efficacy of *Lactobacillus* during sourdough bread fermentation to degrade gluten peptides further than typical processing procedures [102], no therapeutic outcome was detected when α-gliadins processed through sourdough fermentation were exposed to TG2 [103]. However, orally administered enzyme supplements such as *Aspergillus niger* prolyl endopeptidase (AN-PEP) and two proteases from *Sphingomonas capsulate* (ALV003; Latiglutenase) have demonstrated prevention of histopathological changes upon gluten challenge in clinical trials [104,105]. Other enzyme supplements include PvP001, PvP002 and PvP003, which have been investigated in Phase 1 trials for safety and tolerability.

#### 4.2.2. Sequestration

In contrast to the method of assisting complete breakdown of gliadin, inhibition of luminal processing has also been shown to be an effective method for reducing gliadin immunogenicity. This has been demonstrated by both natural and synthetic agents.

Neutralization of gliadin proteins has been achieved in vivo using an anti-gliadin IgY antibody (“AGY”) derived from egg yolk powder, which has the legal status of being “Generally Recognized as Safe”. Oral administration of this powder concurrently with gluten challenge decreased patient-reported symptoms, anti-TG2 antibodies and intestinal permeability as measured by lactulose-mannitol excretion ratios in a small-scale clinical trial. Complexation of gliadins by IgY is thought to prevent the celiac disease immune response by preventing absorption of the proteins into the bloodstream [106,107].

This sequestration effect has also been achieved by oral administration of a synthetic polymer of hydroxyethyl methacrylate-styrene sulfonate, known as poly(HEMA)-co-SS, or commercially as BL-7010 [108]. BL-7010 interacts with α-gliadin at both gastric and intestinal pH levels, disrupting the enzymatic hydrolysis of gliadins and preventing the formation of immunogenic and cytotoxic peptides [10,109]. Upon interaction, BL-7010 also elicits a structural change on α-gliadin peptides [110]. The biological implications of these interactions have been demonstrated in vitro with two cell culture lines, in vivo with transgenic HLA-HCD4/DQ8 gluten-sensitized mice and ex vivo with duodenal biopsies from celiac disease patients. The protective effects of BL-7010 against gliadin-mediated intestinal damage were demonstrated by maintenance of ZO-1 localization along the lateral membrane of Caco-2/15 cells [9]. These findings were further supported by the decrease in gliadin-mediated horseradish peroxidase-flux in gluten-sensitized mice in the presence of BL-7010, which preceded the attenuation of the gliadin-mediated immune response as measured by decreased IEL counts and the absence of lesions in the small intestinal mucosa [9]. A similar study measured the production of anti-gliadin IgA, showing that BL-7010 also improved celiac-related serology, and ex vivo testing of biopsy specimens showed decreased secretion of TNF-α and IL-10 in the presences of the polymer as well [10].

## 5. Polyphenols as Therapeutics for Chronic Inflammatory Diseases

A research area that remains neglected in the treatment of celiac disease is the potential impact of dietary supplements and naturally-derived compounds on gluten digestion and processing. Of interest are polyphenols, which have been shown to have anti-inflammatory properties within the context of chronic diseases of the GI tract and anti-nutritional properties with respect to proteins.

**Figure 1:**
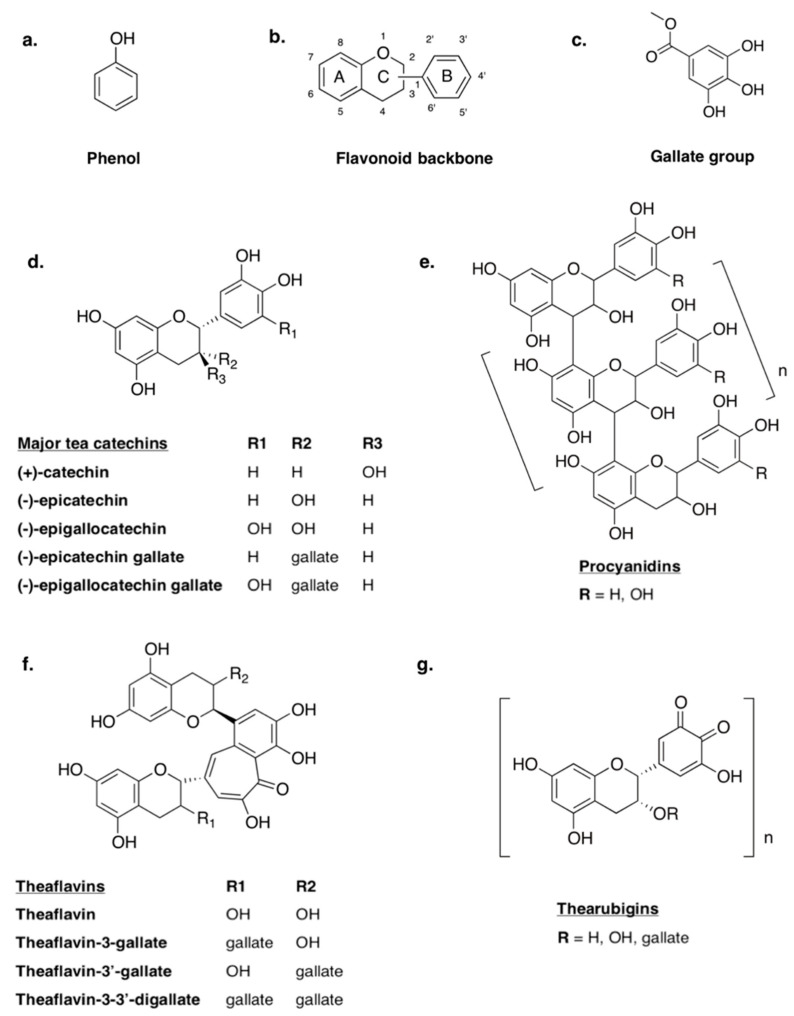
Structures of basic flavonoids and polymerization products: (**a**) a basic phenol, (**b**) the common flavonoid backbone, (**c**) a gallate group, (**d**) major tea catechins, (**e**) the standard molecular structure of B-type procyanidins, (**f**) major tea theaflavins and (**g**) the standard molecular structure of thearubigins.

Polyphenols are a structurally diverse class of secondary metabolites produced by plants to aid non-growth processes such as defense against external stressors, hormone release and signaling within the plant during ripening [111]. Ubiquitous in plants, polyphenols have been widely studied with regard to their potential impact on human health. While the consumption of polyphenols is often associated with health benefits, their ability to inhibit the absorption of nutritive compounds has also been explored as a negative characteristic. After a brief introduction to polyphenols from a chemical standpoint (Figure 1), this section will focus on the anti-nutritional mechanisms and biological impact of polyphenols within the context of human health, as well as applications of this concept that have already been explored with respect to food hypersensitivities.

Polyphenols are organic compounds composed of multiple phenol groups (Figure 1a). Some can be loosely classified as biopolymers, especially in the case of lignins and tannins, which tend to have MWs between 3000 and 20,000 g/mol [112]. Other classes of polyphenols include phenolic acids (e.g., benzoic and cinnamic acids), polyphenolic amides (e.g., capsaicinoids), flavonoids and unclassified non-flavonoids (e.g., ellagic acid, curcumin). Flavonoids are a broad group of polyphenols that are distinguished by their structure—a C6-C3-C6 backbone with two phenolic rings (Figure 1b) [113]. While most polyphenols are highly conjugated and feature multiple phenolic hydroxyl groups and galloyl groups (Figure 1c), variations in structural conformation dictate differences in the classification and functional characteristics. For this reason, flavonoids can be further broken down into sub-classifications. One such class of flavonoids is flavonols, also known as catechins (Figure 1d). All catechins feature two phenolic rings (A and B) linked by a dihydropyran heterocycle, and can be differentiated from one another by isomeric configuration, hydroxylation and the substitution of galloyl groups within the structure. Catechin monomers can also condense to form larger molecules such as the polymeric procyanidins (PCs), dimeric theaflavins (TF) and thearubigins (Figure 1e,f,g) [113].

### 5.1. Protein–Polyphenol Interactions

Polyphenolic interaction with proteins has been well-characterized, particularly with respect to the role that these interactions play in food quality. The ability of polyphenols to contribute to important qualities such as color, flavor and mouthfeel require an understanding of their physicochemical interactions with other compounds in the food system being studied and with the consumer. Tea provides a simple examples for how this phenomenon can affect a product. When milk is added to tea, the tea polyphenols in solution can become bound by milk proteins, which alters polyphenol bioavailability and, thus, the nutritional value of both proteins and polyphenols to consumption [114].

Protein–polyphenol interactions can occur via covalent or non-covalent mechanisms. Covalent interactions typically occur between functional groups on the proteins and phenolic oxidation products (i.e., quinones), which can occur naturally or during processing [115]. Non-covalent interactions between proteins and polyphenols often involve flavonoids, although the structural diversity of flavonoids as a class of compounds introduces complexity to the understanding and characterization of interactions.

Protein structure can be used to predict the characteristics of interaction between proteins and polyphenols. Structurally-defined proteins will often display specific binding pockets whereas non-specific interactions are observed with proteins that have only secondary structural motifs [116]. In either case, non-covalent interactions are the driving force of interaction including van der Waals interactions, π stacking and hydrogen bonding. These interactions have been studied and modeled extensively in relation wine astringency and polyphenolic interaction with salivary proteins, which are rich in proline. Proline-rich proteins have high affinities for polyphenols, facilitated by the structural conformations that occur with high frequencies of proline. The first characteristic is the pyrrolidine ring formation of the proline functional group, which allows for π stacking with polyphenolic rings. Proline also induces an extended/disordered structure, often featuring PPII helices. This type of secondary structure allows increased accessibility to the protein backbone for interaction with polyphenols [117]. These interactions can induce structural changes upon the protein, which in turn can affect protein function. The interactions that occur between proline-rich proteins and polyphenols result in the formation of aggregates, which can precipitate from solution [118,119].

### 5.2. Anti-Inflammatory Properties of Polyphenols

Epidemiological evidence suggests that polyphenols can beneficially impact human health, demonstrating anti-inflammatory, anti-carcinogenic and anti-obesity properties in vitro and in vivo. Often contributing to each of these is the antioxidative capacity of flavonoids. As a class of compounds, polyphenols have the ability to both scavenge free radicals and prevent radical formation, although antioxidative potential can vary between compounds and applications [120]. Epigallocatechin gallate (EGCG), a flavonoid found in tea, has been widely studied for its antioxidative properties, which have been shown to be matrix dependent in vitro [121]. Structurally, EGCG differs from other catechins as it features an ortho-trihydroxyl group in the B ring and a galloyl moiety [122]. The trend for di-vs. trihydroxy substitutions to affect redox potential has been reported for other catechins, showing that structurally similar compounds such as catechin, epicatechin and epigallocatechin possess similar redox potentials [123].

The bioactivity of flavonoids and other polyphenols is not limited to the neutralization or prevention of free radicals, or protection against oxidative damage. The role that flavonoids can play in cell signaling and anti-inflammatory compounds has been investigated in vitro and in vivo through their ability to induce or inhibit enzymes that play important roles in cell maintenance [124]. Flavonoids have been shown to modify the activity of enzymes associated with oxidative stress, such as inducible nitric oxide synthase (iNOS), lipoxygenases, and cyclooxygenases. Endogenous enzyme iNOS is responsible for the production of nitric oxide (·NO), which is a highly reactive compound capable of oxidizing cellular proteins and DNA [124,125] and whose activity has been shown to be decreased by tea flavonoids [124,126,127]. Similarly, the ability of flavonoids to compromise the activity of lipoxygenases and cyclooxygenases has been used to explain a potential mechanism for the anti-cancer properties of flavonoids. Enzymes 15-lipoxygenase and COX-2 have both been found to be upregulated in colon cancer cells compared to normal epithelial cells, but this increase in activity can be reversed in vitro in human mucosal tissue, and in vivo in mice and rats given tea flavonoids in their diet in the form of green tea extract added to their drinking water [124,128].

The anti-inflammatory capabilities of flavonoids have been investigated with respect to a variety of disease states. Obesity is characterized by chronic, low-grade inflammation, which plays a role in the development of other diseases such as type-2 diabetes and cardiovascular disease. EGCG has the ability to decrease tumor necrosis factor (TNF)-α signaling in vitro and in vivo. In the development of atherosclerosis, TNF-α induces endothelial cell secretion of monocyte chemoattractant protein (MCP)-1, which is associated with the development of atherosclerotic plaques. Pre-treatment of porcine endothelial cells with EGCG prevented TNF-α-induced MCP-1 upregulation [129]. In liver tissue of obese mice, EGCG prevented steatohepatitis and improved insulin resistance by similarly decreasing TNF-α, linked to an overall suppression of NF-*κ*B signaling [130]. The suppression of NF-*κ*B signaling by EGCG also influences the immune system in obese individuals, reducing the impact of the obesity-induced inflammatory state by promoting the proliferation of regulatory T cells and secretion of anti-inflammatory cytokine IL-10 [131].

### 5.3. Anti-Nutritional Properties of Polyphenols

Despite their numerous health benefits, polyphenols are sometimes referred to as “anti-nutrients”, as polyphenol-rich diets have been associated with reduced absorption and digestibility of micro- and macronutrients. Underlying mechanisms driving the anti-nutritional effects of polyphenols in the diet include digestive enzyme inhibition and protein sequestration.

The inhibition of digestive enzymes by extracts of plant products including grape seed, berries and tea has been demonstrated in vitro. This effect does not only apply to endogenous enzymes; bacterial enzymes can also be affected [132]. Enzyme inhibition has been shown to be a therapeutic target for obesity and metabolic syndrome, as decreased absorption of fat and carbohydrates can result in improved health outcomes. For this reason, lipase and amylase inhibition by polyphenols and polyphenol-rich diets have been examined extensively with obesity-related outcomes [133,134,135,136].

Inhibition of digestive proteases has also been studied, with polyphenols showing inhibitory effects on trypsin activity, while conflicting data exist with regard to pepsin [137]. In vitro, green tea extract and its constituent compounds have been shown to inhibit trypsin activity [138,139]. Pepsin, on the other hand, has been shown to be inhibited [139], unaffected [138] and even activated [140] in the presence of polyphenols in three different studies. Based on both empirical data and in silico modeling studies, the inhibition of digestive enzymes by polyphenols appears to be due to direct interactions between the polyphenols and the enzymes, as polyphenols have a natural propensity to interact with proteins [141]. However, when the target substrates of an enzymatic reaction are also proteins, the possibility that polyphenols sequester the substrate from enzymatic catalysis cannot be ruled out.

### 5.4. Therapeutic Applications of Polyphenols

Polyphenols have been shown to influence the immune system within the context of food-related and digestive diseases both directly, as immunomodulators, and indirectly as sequestrants of allergenic proteins. The immunomodulatory activity of polyphenols is demonstrated by their ability to disrupt cell signaling pathways, modify cytokine production and concomitantly affect T cell proliferation and migration. These characteristics have been explored extensively within the context of IBD as well as food allergies, both of which share overlapping characteristics with celiac disease.

#### 5.4.1. Polyphenols as Therapeutic Agents for IBD

In general, polyphenols have been implicated as potentially beneficial in the mediation of IBD, which includes ulcerative colitis (UC) and Crohn’s disease. Both of these diseases are characterized by chronic inflammation within the gastrointestinal tract. While UC primarily affects the colon, Crohn’s disease can manifest along the entirety of the gastrointestinal tract, most commonly appearing in the terminal ileum [142]. A key difference between celiac disease and IBD is IBD’s lack of a single, specific environmental/dietary trigger. However, IBD shares a number of similarities with celiac disease not only pertaining to what is known about it, but also to what remains unknown. Similar to celiac disease, UC and Crohn’s disease are both thought to develop as a result of both genetic and environmental factors. Although those environmental factors are presently undefined, hypotheses include drug exposure, the microbiome and stress. Furthermore, the pathogenesis of each of these conditions feature both innate and adaptive immune responses in the intestinal mucosa and present similar symptoms and physiological effects such as gastrointestinal discomfort, inflammation of the gastrointestinal tract and intestinal barrier permeability [142,143]. IBD treatment also mirrors that of celiac disease, as it is focused on symptom management and disruption of the immune response via administration of anti-inflammatories and immunosuppressants, although alternative and dietary therapies are currently being explored. Among them are pre- and probiotics, as well as polyphenol supplementation [144].

Studies on the potential for polyphenols as a complementary therapy for IBD have shown that the anti-inflammatory and immunomodulatory effects of tea flavonoids observed in liver tissue and endothelial cells—inhibition of COX-2, decreased NO production, NF-*κ*B suppression—are also observed in the intestinal mucosa, resulting in overall amelioration of the inflammatory state of IBD [127,143,145]. Histological damage such as crypt hyperplasia and IEL infiltration can also be prevented by flavonoid treatment [143,146]. Decreased IEL infiltration as a result of flavonoid treatment is demonstrative of the therapeutic effect that flavonoids can have on the immune dysregulation that is associated with IBD. By suppressing NF-*κ*B, tea polyphenol EGCG has been shown to reduce TNF-α expression by peritoneal macrophages [145,147], and IEC lines Caco-2 and IEC-6 [143,146,147]. EGCG has also been shown to modify cytokine secretions in Caco-2 cells, reducing proinflammatory cytokines IL-6 and IL-8 [148], which play a role in intestinal barrier permeability and IEL homing, respectively [149,150].

Clinical trials focusing on the impact of dietary polyphenols on IBD demonstrate the therapeutic efficacy of these compounds in practice. The recent European Prospective Cohort (EPIC) study delineated an inverse association between the intake flavones and incidence of Crohn’s disease over the course of the study [151]. Intervention studies on the effect of mango (*Mangifera indica* L.) polyphenols on IBD have shown decreases in Simple Clinical Colitis Activity Index over the course of 8 weeks, along with decreases in plasma IL-8, growth-related oncogene and granulocyte macrophage colony-stimulating factor [152]. Similarly, oral administration of anthocyanin-rich bilberry extract led to 6 out of 10 patients in a small clinical trial reaching remission. In these patients, reduced colonic expression of STAT1 and IFN-γ receptors 1 and 2 were observed along with reduced plasma levels of proinflammatory cytokine MCP-1 and increased plasma levels of anti-inflammatory cytokine IL-10 [153]. These changes in biomarkers and immune cell signaling are consistent with the aforementioned findings of in vitro and ex vivo studies, which outline decreased IEL infiltration in the intestinal mucosa as a potential protective mechanism of polyphenols against IBD.

#### 5.4.2. Polyphenols as Mediators of Allergic Responses

While not a classic food allergy, celiac disease is similar to a food allergy in that it is stimulated by a food-based protein antigen. The efficacy of polyphenols in the mitigation of allergic responses has been investigated as both part of food matrices and as natural treatments for protein-stimulated inflammatory responses.

Apple polyphenols have shown anti-allergenic effects by dose dependently suppressing the expression of MHC class II molecules when tested in ovalbumin-stimulated dendritic cells via upregulation of the membrane associated ring-CH type finger 1 (MARCH1) gene, which downregulates surface molecules. However, TNF-α was upregulated and IL-10 was downregulated [154], demonstrating that these particular compounds are not protective against inflammation. In a similar study, apple polyphenols prevented IgE-mediated allergic responses to ovalbumin in mice, including anaphylaxis. Immune signaling in the gut was affected by consumption of apple extract, as IL-5, IL-13 and CCL11 were all downregulated. The allergenicity of ovalbumin was greatly reduced in the presence of apple polyphenols according to an IgG enzyme-linked immunosorbent assay, suggesting that the modification to the immune signaling pathway may be due to sequestration of the allergenic protein [16].

The use of polyphenols as protective agents against food allergens has been explored most notably with the creation of hypoallergenic peanut butter [17,19,155,156]. In these studies, peanut allergens Ara h 1 and Ara h 2 were complexed with caffeic, chlorogenic or ferulic acid, which resulted in significantly reduced IgE binding [19]. A follow-up study used tannic acid as a precipitation agent, removing Ara h 1 and Ara h 2 from solution and similarly preventing recognition by IgE [155]. Further investigation of this topic has led to the development of an edible peanut butter matrix with reduced allergenicity containing polyphenols from a variety of plant sources including cranberries, cinnamon and green tea [17,156]. The polyphenol-fortified matrices demonstrated reduced allergenicity by way of reduced IgE binding, basophil activation and mast cell degranulation, but the stability of the complexes and maintenance of hypoallergenicity was dependent upon the type of polyphenol used [156].

The mechanistic explanation for decreased binding and recognition of allergenic proteins has been explored using an array of analytical techniques and purified forms of the allergenic proteins. Studies characterizing the interactions between EGCG and peanut allergens Ara h 2 and Ara h 6 demonstrate binding specificity upon formation of protein–polyphenol complexes. Additionally, binding elicits a structural change on the allergens, modifying the frequencies of α-helices and β-sheets within each. In silico analyses revealed potential binding sites on each Ara protein, which is useful in predicting the impact of binding on epitope recognition by immune cells [18]. The mechanistic findings of this study could potentially help explain the effects of reduced allergenicity in the previous peanut allergen study; however, the absence of mechanistic data in the first and biological application in the second prevent the drawing of clear conclusions.

## 6. Polyphenols as a Prospective Therapy for Celiac Disease

Polyphenols have demonstrated efficacy in protection against diseases with similar etiologies as celiac disease in vitro and in vivo due to their abilities to both interact with immunostimulatory proteins and to mediate inflammatory responses. Recently, a limited number of studies have investigated how these effects may translate to a celiac disease model, building a strong foundation for the therapeutic potential of polyphenolic supplementation as a complementary or alternative treatment for celiac disease. While interactions between gliadin and polyphenols have been demonstrated in a variety of matrix-specific contexts over the last decade, the first evidence of biological efficacy for polyphenols in the treatment of celiac disease was delineated in 2017. In this seminal study, gliadin–polyphenol complexes were characterized and shown to be stable throughout digestion and prevent gliadin-mediated inflammation and permeability in vitro [11]. This work has since been corroborated and expanded upon to reveal a variety of potential mechanisms by which polyphenols may exert protective effects against celiac disease symptoms and pathogenesis.

**Figure 2:**
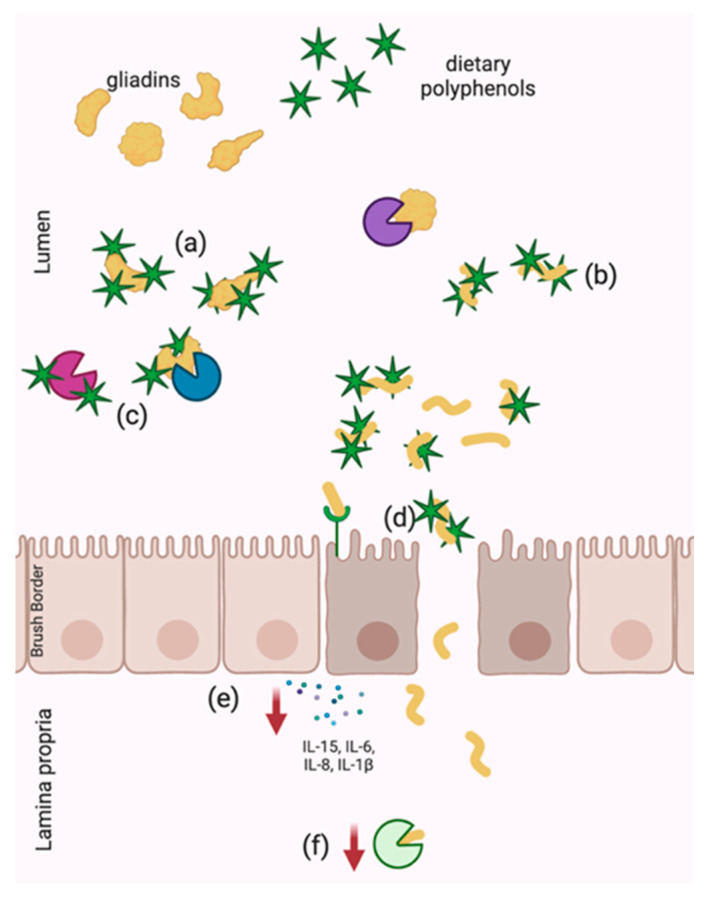
Demonstrated mechanisms of celiac disease pathogenesis disruption by dietary polyphenols including (**a**) physical sequestration of native gliadins and (**b**) hydrolyzed gliadins, (**c**) digestive enzyme inhibition, (**d**) improved barrier integrity and decreased paracellular transport of gliadins, (**e**) anti-inflammatory activity and (**f**) TG2 downregulation. Arrows represent decreased expression.

The potential benefits of polyphenol supplementation for individuals with celiac disease have been shown to stem from both the anti-nutritional and anti-inflammatory properties of polyphenols. Polyphenols are not only potent anti-inflammatory compounds, as discussed with respect to IBD, but they also possess the ability to interact with proteins, allowing for direct sequestration of gliadin and disruption of pathogenesis through inhibition of digestive enzymes. The protective effects of polyphenols within the context of celiac disease that have been demonstrated to date are summarized in Figure 2.

### 6.1. Gliadin–Polpyhenol Interactions

Gliadin–polyphenol interactions are dependent on a variety of factors including the specific nature of gliadins, the structure of polyphenols and the reaction matrix. Gliadin–polyphenol complexes that exist naturally within food matrices [157,158] or those which were developed specifically with ingredient functionality in mind [159,160,161,162] tend to feature lower pH environments and demonstrate the formation of soluble complexes driven by non-specific, non-covalent interactions. Specifically, investigations into gliadin-anthocyanin complex formation have been carried out only between pH 2.5 and 4.3 as these conditions are translatable to wine, where gluten has been applied as a processing aid, and in order to favor gliadin solubility and polyphenol stability [159,160,161,162].

The trend of non-specificity when it comes to interactions between gliadins and polyphenols has been shown to be consistent across pH levels and polyphenolic structures, likely due to the absence of a “true” (i.e., crystallizable) gliadin structure, though binding conformation and affinities are affected by polyphenol structure. Structural characteristics of polyphenols that influence binding include molecular weight, degree of galloylation and degree of polymerization. Saturation transfer difference NMR has shown that while increasing each of these characteristics in a given polyphenols tends to increase binding affinity to gliadins, increasing degree of galloylation and/or polymerization tends to be more influential than increasing molecular weight [11,163,164]. The influence of degree of polymerization of a given procyanidin and galloylation of flavonoids on interactions with gliadin offer interesting insights towards the application of these compounds from a therapeutic perspective. Studies into the specific molecular mechanisms have shown that that phenolics with structures that branch out with greater potential interaction sites have greater affinity for interaction with gliadins, suggesting that these compounds (i.e., EGCG, procyanidins) should be a focal point in the development of nutraceutical approaches for mitigating celiac disease symptoms. However, it must be noted that the degree to which many of these studies can be translated to human health is limited, as the reaction systems are all static, with neutral pH and purified reactants.

Direct interactions of polyphenols with gliadins have the potential to influence celiac disease pathogenesis by sequestering gliadins in order to inhibit recognition and absorption by the body as well as modifying the structure of immunostimulatory gliadin peptides, which may prevent their ability to bind to key receptors and enzymes.

#### 6.1.1. Physical Sequestration of Gliadins

The formation of gliadin–polyphenol complexes can be beneficial in protecting against celiac disease if these complexes allow sequestration of the protein and prevent it from interacting in the celiac disease milieu. Physical sequestration of gliadins via complexation has been shown to be effective in clinical trials with the synthetic, non-bioavailable polymer BL-7010 [10,108]. Early mechanistic studies of this polymer showed that it was able to precipitate gliadins from solution, forming insoluble complexes stable across digestive pH levels and prevented an immune response when presented concomitantly with gliadin to gluten-sensitized mice [10,110]. Via the aforementioned protein–polyphenol interactions, a similar sequestration effect has been demonstrated in vitro using green tea polyphenols as a biological sequestrant [12].

Green tea polyphenols (including EGCG) are able to precipitate both native and pepsin-trypsin-digested gliadins via the formation of insoluble complexes throughout in vitro digestion processes, resulting in an overall reduction in total protein levels of a sample digest [12]. Similar to the effects observed with BL-7010, this complexation may render gliadin inaccessible to the body and thus prevent uptake and a subsequent immune response. The ability of polyphenols to precipitate gliadins at any point in the digestion process is of significant interest in therapeutic development, as this may potentially allow for “scavenging” of gliadins by polyphenols at various points throughout digestion and eliminate a requirement for administration of polyphenols at a specific time in relation to gluten challenge.

Interactions between a uniquely immunostimulatory gliadin, α_2_-gliadin (57–89), and EGCG have also been characterized at pH 2.0, 6.8 and 7.2, demonstrating similar formation of insoluble complexes and interactions between EGCG and specific proline and glutamine residues, which play an important role in antigen presentation in celiac disease pathogenesis [13]. The 33-amino acid peptide α_2_-gliadin (57–89) has been extensively studied for its dominant role in stimulating the celiac disease immune response. With the sequence LQLQPFPQPQLPYPQPQLPYPQPQLPYPQPQPF, this peptide features six overlapping epitopes for enzymatic binding and immune recognition [21]. While the effects of EGCG on antigen presentation of α_2_-gliadin (57–89) has not yet been investigated, these findings suggest that EGCG may physically block immunostimulatory epitopes on this peptide in a phenomenon known as “epitope masking”. This phenomenon has been demonstrated within the context of food allergies. Perot and others showed that pre-treatment of native gliadins with polyphenol extracts from cranberry and apple reduced gliadin recognition by IgE and IgG antibodies, which are relevant in wheat allergy [165]. This promising finding may translate to celiac disease if polyphenol complexation can prevent events such as deamidation, antigen presentation and anti-gliadin IgA recognition.

#### 6.1.2. Structural Change

A key feature in epitope masking and disruption of protein-mediated immune responses as a result of physical sequestration is thought to be structural change. In many cases of food allergy mitigation with polyphenols, structural changes in allergenic proteins have been predicted or observed [18,166,167]. Gliadin is a unique protein in its absence of a consistent, singular structure. Instead, gliadin is characterized by repeat motifs and PPII helices. Structural changes in gliadins as a result of interaction with polyphenols have been noted in both native and hydrolyzed gliadins using a variety of analytical methods including Raman spectroscopy, NMR and circular dichroism [13,161,168].

Interactions between EGCG and a_2_-gliadin (57–89) have been shown to cause the protein to transition from disordered to ordered by increasing relative helicity of the protein at a range of digestive pH levels [13], a finding that has been corroborated in a similar peptide using nuclear Overhauser effect spectroscopy at neutral pH [169]. This structural change may have significant biological relevance, as celiac disease pathogenesis is driven in part by structural recognition of gliadin’s repeat motifs. In antigen presentation, MHC class II molecules demonstrate affinity for polyproline II helical peptides and the P6 binding pocket of DQ2 preferentially and tightly binds proline residues [170]. These structural preferences of HLA-DQ2 make the proline and glutamine-rich fragments of gliadin that transcend the brush border, which also tend to form polyproline II helices, attractive targets for binding and forming MHC class II complexes [171]. Further studies are required to determine the role of structural modification of gliadins on immune signaling versus epitope masking within the context of celiac disease.

### 6.2. Inhibition of Gliadin Digestion

The digestion of gliadins into smaller, immunostimulatory fragments in the lumen of the small intestine is a critical first step in celiac disease pathogenesis. Prevention of this step has been shown to reduce the immune response to gliadin in vivo [108]. Inhibition of digestive enzymes including pepsin and trypsin is a known biological effect of many dietary polyphenols, contributing to their anti-nutritional characteristics. The influence of enzyme inhibition on gliadin digestion within the context of celiac disease has been explored in vitro, where supplementation with green tea extract caused a decrease in the formation of low molecular weight gliadins in addition to the aforementioned precipitation of protein from solution. Beyond direct precipitation of native and digested gliadins, green tea polyphenols have been shown to dose dependently inhibit both pepsin and trypsin [12]. While this provides a promising option for celiac disease disruption, widespread digestive enzyme inhibition may be problematic for the intake of other, non-immunostimulatory proteins.

### 6.3. Protective Effects of Polyphenols Towards Celiac Disease In Vitro

Despite a large number of studies focusing on the interactions between gliadin proteins and peptides with polyphenols, only a limited number have investigated the biological implications of these interactions and all of these studies have used an in vitro model of the disease. The Caco-2 monolayer model is a simplified model of the intestinal barrier wherein human colonocytes are seeded onto a semi-permeable membrane and grown to confluence. This cell model expresses characteristics of the human small intestine including the brush border enzymes, tight junction proteins and microvilli [172], and has been used extensively to study celiac disease pathogenesis [31,46,173,174]. Each of the studies that have tested the efficacy of polyphenols as a therapeutic option for celiac disease have used Caco-2 cells as an in vitro model, either as confluent monolayers [12,168,169] or a traditionally seeded culture [175]. The findings from these studies are discussed below and summarized in Table 2.

#### 6.3.1. Initiation of Intestinal Permeability

As previously discussed, the initiation of intestinal permeability is an early step in celiac disease pathogenesis leading to the adaptive immune response mucosal damage. The ability of polyphenols to prevent intestinal permeability has been demonstrated in the Caco-2 monolayer model by measuring transepithelial electrical resistance (TEER). The decrease in TEER, or increase in intestinal permeability, observed upon treatment of cells with pepsin-trypsin-digested gliadin can be prevented by concurrent treatment with green tea extract [12]. The precise mechanism for this increase/stabilization of TEER is not clear, as green tea extract increased TEER over baseline and control levels in both the presence and absence of gliadin. While gliadin sequestration and prevention of zonulin production is a potential mechanism, alternative mechanisms may also exist, including the upregulation of glucagon-like peptide-2, a hormone associated with barrier integrity that is not directly associated with celiac disease [176].

#### 6.3.2. Transport

Transport of gliadins from the lumen to the lamina propria follows the initiation of intestinal permeability in the celiac disease milieu. Procyanidins B3 and C2 have both been shown to effectively prevent the transport of a gliadin peptide from the apical to basolateral compartment of the Caco-2 monolayer model over the course of 3 h [169], and treatment with EGCG corroborated the previously discussed mediation of TEER by similarly preventing gliadin transport across the monolayer [168]. In each of these studies, a mechanism for transport prevention remains undefined, though multiple possibilities exist. The peptide used in these studies is not typically associated with tight junction disruption and the absence of TEER data makes it unclear whether transport is affected by a mechanism such as sequestration where permeability is actively prevented, or if perhaps permeability is increased anyway, but the greater hydrodynamic radii of gliadin–polyphenol complexes were too large for paracellular transport. Furthermore, as discussed for the initiation of intestinal permeability, all of the polyphenols tested in these studies have the potential to improve barrier function independently of celiac disease-specific mechanisms, which may play a role in reducing gliadin transport [177].

#### 6.3.3. Amelioration of Gliadin-Mediated Inflammation

The celiac disease inflammatory response which drives mucosal damage and the overactive immune response associated with the condition is the most popular target for celiac disease interventions. The innate immune response is associated with the production of pro-inflammatory cytokines associated with mucosal damage and celiac disease “hallmark” IL-15 [39,40]. Following deamidation by TG2, gliadin goes on to activate the adaptive immune response and subsequent release of other pro-inflammatory cytokines including IFN-γ, IL-1β, IL-6, IL-8 and TNF-α [57,178,179,180,181,182].

Mediation of the celiac-related inflammatory response by polyphenols has been demonstrated in Caco-2 cells with both green tea extract [12], which is rich in catechins including EGCG, and a cocoa extract enriched for procyanidin B2 [175]. Green tea extract prevented gliadin-mediated increases in IL-6 and IL-8 [12]. IL-6 has been associated with gut barrier integrity [147], and was thus likely linked to the observed enhancement of barrier integrity in the same study. IL-8, on the other hand, is associated with the recruitment and activation of IELs [183], which cannot be observed in a system of Caco-2 cells alone, but suggests that this treatment may be beneficial in previously unrealized ways in a more complex model of celiac disease.

The procyanidin B2-rich cocoa extract applied to Caco-2 cells in the presence of bioactive gliadin peptide p31-43 intervened in the celiac-specific inflammatory cascade, decreasing secretion of IL-15, IL-1β, IL-6 and IL-8 in comparison to p31-43 alone. The extract was also effective in decreasing the inflammatory response when stimulated by IFN-γ [175], though relative comparisons of cytokine levels between antagonist groups (i.e., IFN-γ vs. p31–43) do not allow the direct comparison that would be needed to determine whether the efficacy of the cocoa extract resulted from gliadin–polyphenol interaction or a standard anti-inflammatory response.

Most striking from this study was that the inhibition of the gliadin-mediated inflammatory response resulted in a significant decrease in TG2 production by Caco-2 cells [175]. TG2 is ubiquitous in the human body and has been implicated in a variety of biological processes including extracellular matrix formation, cell differentiation and signal transduction, though its most common role in the intestinal mucosa is tissue repair [184,185]. Notably, it has shown specific affinity for glutamine residues located one amino acid away from a proline residue—a pattern that is heavily conserved within the proline and glutamine rich digestive products of gliadin [52]—and plays a key role in increasing the binding affinity of MHC class II receptors to gliadins via deamidation. Though the effect of the cocoa extract on TG2 appeared to be greater when the cells were stimulated by IFN-γ, suggesting direct interaction with the cells rather than protein sequestration, polyphenols preventing TG2 activity within the context of celiac disease presents an exciting new therapeutic consideration in the development of this nutraceutical approach to treatment.

## 7. Conclusions

Polyphenols are widely known for their varied effects on human health, including anti-inflammatory and anti-nutritional activities. These characteristics make polyphenols a uniquely interesting therapeutic option for protein-stimulated inflammatory diseases such as celiac disease. Significant progress has been made in the understanding of the potential for polyphenols to be used as a therapeutic agent for celiac disease over the last decade, though the studies completed to date are not without limitations. Satisfactory in vivo studies testing the efficacy of these treatments have yet to be completed, and many of the in vitro studies available do not fully delineate intervention mechanisms, as proof of principle is still an important objective in each system with different polyphenolic profiles and gliadin sources. Furthermore, the versatile bioactivity of polyphenols lends them to a variety of potential beneficial effects within celiac disease pathogenesis, many of which will not be discovered until more complex disease models are used.

**Figure 3:**
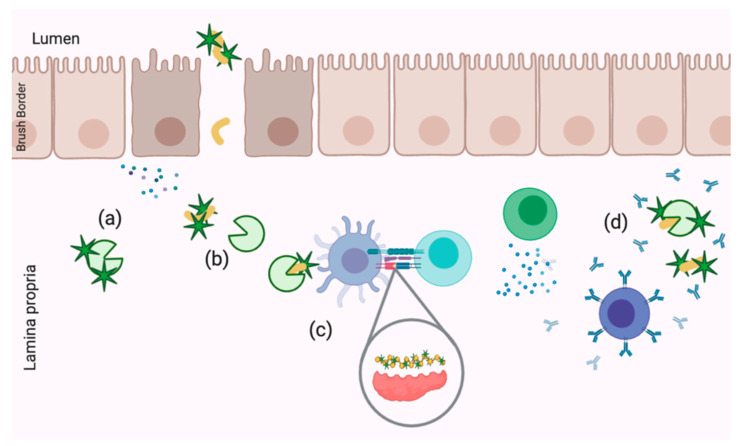
Demonstrated mechanisms of celiac disease pathogenesis disruption by dietary polyphenols including (**a**) TG2 inhibition, (**b**) TG2 epitope masking, (**c**) antigen presentation disruption and (**d**) anti-gliadin IgA epitope masking.

Based on what is known about celiac disease and protein sequestration via polyphenols as a treatment option, more research is needed to determine the impact of structural modification on celiac-specific immune signaling versus classical anti-inflammatory effects of polyphenols. While the idea of polyphenolic sequestration is more novel, the bioactive properties of free polyphenols within the context of celiac disease cannot be overlooked, especially considering their efficacy as anti-inflammatory and antioxidative compounds in chronic diseases with similar etiologies, such as IBD. Potential mechanisms of interest relating to gliadin–polyphenol interactions include decreasing TG2 activity via enzyme inhibition or epitope masking, disruption of antigen presentation and anti-gliadin IgA epitope masking (Figure 3). Each of these potential functions for polyphenols, whether direct of via gliadin sequestration, require disease models which feature both IECs and IELs. However, these questions can also be approached with molecular dynamics and computational modeling.

A nutraceutical therapy for celiac disease treatment is an attractive option for clinicians and patients alike. Polyphenols, ubiquitous to plant-based foods, are already found in a typical diet, and are safely taken as supplements by many. To date, polyphenols are the only common dietary component being explored as a treatment option for celiac disease, in comparison to a myriad of synthetic pharmaceuticals compounds which require extensive testing for safety and tolerance before efficacy can even be approached. While there is a long way to go between the studies described here and true clinical use of this treatment option, the groundwork has been laid for further investigation towards a treatment for celiac disease that is safe, effective and well understood.

## Figures and Tables

**Table 1:** Current and ongoing clinical trials for celiac disease treatments beyond the scope of the gluten-free diet as of December 2020.

Intervention	Outcomes	Identifier and Phase
***Probiotic***		
*Lactobacillus plantarum, Lactobacillus casei, Bifidobacterium breve, Bifidobacterium animalis*	Gastrointestinal symptom improvement	NCT01699191
Pentabiocel (probiotic cocktail)	1°: Changes in celiac symptom index; 2°: Changes in serology, BMI	NCT03857360
*Lactobacilli* culture	Anti-TG2 antibodies	NCT03176095 ^1^
BL NCC 2705	Safety and tolerability	NCT03775499
*L. paracasei, L. plantarum*	1°: Serum antibodies for TG2, GADA, IA-2, IAA, ZnT8a, TPOA; 2°: Concentration of gluten peptides in urine	NCT04014660
*Bifidobacterium infantis*	1°: Gastrointestinal symptom improvement; 2°: Changes in celiac symptom index, fecal microbiome diversity, changes in gluten immunogenic peptides, serology, anthropometric measurements	NCT03271138 ^1^ Phase 2
	1°: Decreased intestinal permeability; 2°: Changes in cytokine profile	NCT01257620 ^1^
***Prebiotic***		
Inulin-type fructans	1°: Decreased intestinal permeability; 2°: Adverse events, short-chain fatty acids, molecular characteristics of fecal microbiota	NCT03064997 ^1^
***Antibiotic***		
Rifaximin	1°: Gastrointestinal symptom improvement; 2°: Decreased small intestine bacterial overgrowth	NCT01137955 ^1^
***Parabiotic***		
*Necator americanus* infection	1°: Histopathology (villous height:crypt depth); 2°: IEL counts over the course of treatment, gastrointestinal symptom improvement, anti-TG2 antibodies	NCT02754609 ^1^ Phase 1b
	1°: Histopathology (villous height:crypt depth); 2°: IEL count, Marsh score, anti-TG2 antibodies	NCT01661933 ^1^ Phase 1/2
	1°: Marsh score; 2°: PBMC gluten recognition, T cell proliferation, cytokine profiles	NCT00671138 ^1^ Phase 2
***Anti-Inflammatory***		
PTG-100	1°: Histopathology (villous height:crypt depth); 2°: Changes in anti-TG2, antiDGP antibodies, CD3-positive IEL density, changes in celiac symptom index	NCT04524221 Phase 1
***Anti-IL-15 Antibody***		
Hu-Mik-β-1	Safety in celiac disease patients	NCT01893775 ^1^ Phase 1
CALY-002	Safety and tolerability	NCT04593251 Phase 1
PRV-015	1°: Celiac disease patient-reported outcomes; 2°: IEL density, safety and tolerability, pharmacokinetic analysis, anti-PRV-015 antibodies	NCT04424927 Phase 2
AMG 714	1°: Reduction in IELs; 2°: Improvement of histopathology, gastrointestinal symptom improvement	NCT02633020 ^1^ Phase 2a
TM-β1	1°: Attenuation of mucosal injury; 2°: Decreased IEL infiltration, attenuation of anti-gliadin and anti-TG2 serum antibodies, gastrointestinal symptom improvement	NCT02637141 ^1^ Phase 2a
***Immunomodulators***		
Cathepsin S inhibitor (RG 7625)	1°: Decreased number of responses to gluten challenge; 2°: Number of patients with adverse events, attenuation of anti-TG2 and anti-gliadin antibodies, improved lactulose/mannitol ratio, decreased circulating white blood cells, concentrations of CD74 B cells	NCT02679014 ^1^ Phase 1
Vedolizumab	Histopathology	NCT02929316 ^2^ Phase 2
Nexvax2	Safety and tolerability	NCT00879749 ^1^ Phase 1
	1°: Safety and tolerability, intervention bioavailability; 2°: Pharmacodynamic measures, pharmacokinetic analysis	NCT03543540 ^1^ Phase 1
	1°: Safety in celiac disease patients; 2°: Gastrointestinal symptom improvement, plasma cytokine levels	NCT02528799 ^1^ Phase 1
	1°: Celiac disease patient-reported outcomes; 2°: Pharmacodynamic measures of immune activation, gastrointestinal symptoms, safety and tolerability	NCT03644069 Phase 2
Vercirnon (CCX282-B)	1°: Histopathology (villous height:crypt depth); 2°: Small intestinal inflammation, gluten-induced celiac-type serology, gastrointestinal symptom improvement	NCT00540657 ^1^ Phase 2
Short-course steroids (Prednisolone)	1°: Gastrointestinal symptom improvement, histopathology; 2°: Maintenance of histopathology	NCT01045837 ^1^ Phase 2/3
KAN-101	1°: Safety and tolerability; 2°: Pharmacokinetic analysis	NCT04248855 Phase 1
TIMP-GLIA	1°: safety and tolerability, clinically significant change in physical examination, vital signs; 2°: Pharmacokinetic analysis	NCT03486990 ^1^ Phase 1
	1°: Changes in baseline plasma IFN-ɣ; 2°: Changes in gliadin-specific T cell proliferation, cytokine secretion, changes from baseline in T cells, histopathology (villous height:crypt depth), changes in number of IELs, plasma concentration of intervention	NCT03738475 ^1^ Phase 2
TAK-101	1°: Changes in baseline plasma IFN-ɣ; 2°: Safety and tolerability, changes in celiac symptom index, changes from baseline IL-2, pharmacodynamic measures	NCT04530123 Phase 2
***Zonulin Antagonist***		
Larazotide acetate	1°: Safety and tolerance, pharmacokinetic analysis, intestinal permeability, zonulin levels; 2°: Gastrointestinal symptoms Pharmacodynamic measures	NCT00386165 ^1^ Phase 1
	1°: Safety and tolerability; 2°: Intestinal permeability, celiac disease signs and symptoms	NCT00362856 ^1^ Phase 2
	1°: Intestinal permeability; 2°: Composite celiac disease activity index, adverse events	NCT00492960^1^ Phase 2
	1°: Response to gluten; 2°: Attenuation of anti-TG2 antibodies	NCT00889473 ^1^ Phase 2
	1°: Histopathology (villous height:crypt depth); 2°: Safety and tolerability	NCT00620451^1^ Phase 2
	Gastrointestinal symptom improvement	NCT01396213 ^1^ Phase 2b
	Celiac disease patient-reported outcomes	NCT03569007 Phase 3
***TG2 Inhibitor***		
GSK3915393	1°: Safety and tolerability, clinically significant change in vital signs, physical examination; 2°: Pharmacokinetic analysis	NCT04604795 Phase 1
***Dietary Replacement***		
*Triticum monococcum*	1°: Histopathology; 2°: Attenuation of anti-TG2 and anti-endomysial antibodies, gastrointestinal symptom improvement	NCT02220166 ^1^ Phase 2
Microbial transglutaminase-treated wheat flour	1°: Attenuation of anti-TG2, anti-endomysium antibodies, histopathology (villous height:crypt depth, IEL count, Marsh–Oberhuber score); 2°: Gastrointestinal symptom improvement	NCT02472119 Phase 2
Gluten-friendly bread	1°: Changes in serology; 2°: Fecal microbiome diversity, gastrointestinal symptoms, lactulose/mannitol excretion ratio	NCT03168490 ^3^
	Small bowel mucosal density, anti-TG2 IgA EMA and AGA antibody levels	NCT03137862 ^1^
Protalsafe	1°: Lactulose/mannitol excretion ratio, serum zonulin 2°: Fecal microbiome profile, gastrointestinal symptoms, quality of life, changes from baseline in serum cytokines	NCT03483805 ^1^
***Enzyme Supplement***		
*Aspergillus niger* prolyl endoprotease	1°: Histopathology/Marsh score, attenuation of anti-TG2, anti-endomysial and anti-gliadin antibodies; 2°: Presence and activity of gluten-reactive T cells, immunophenotype of lymphocytes, clinical symptoms after gluten challenge	NCT00810654 ^1^ Phase 1/2
Pancreatic enzyme	1°: Gastrointestinal symptom improvement; 2°: Fecal elastase measurement	NCT02475369 ^2^ Phase 4
Proprietary food-grade enzyme blend	1°: Decreased anti-TG2 antibodies; 2°: Decreased anti-endomysial antibodies, decreased celiac-related antibodies in blood, change in symptoms, including skin rash, histopathology improvement	NCT00962182 ^1^ Phase 1/2
ALV003 protease	1°: Clinical symptoms, celiac-specific serology; 2°: Immunotoxic epitope profile of digests	NCT00859391 ^1^ Phase 1
	Safety and tolerability	NCT00626184 ^1^ Phase 1
	Safety and tolerability	NCT00669825 ^1^ Phase 1
	1°: Histopathology, tolerability; 2°: IEL count/phenotype, serological markers	NCT01255696 ^1^ Phase 2
	1°: Histopathology (villous height:crypt depth); 2°: IEL density, safety and tolerability, celiac-specific serology	NCT01917630 Phase 2
	1°: Histopathology, tolerability; 2°: IEL count/phenotype, serological markers	NCT00959114 ^1^ Phase 2a
PvP001, PvP002, PvP003	1°: Safety and tolerability; 2°: Pharmacokinetic analysis	NCT03701555 Phase 1
Latiglutenase	1°: Histopathology; 2°: Gastrointestinal symptom improvement	NCT03585478 Phase 2
	1°: Gastrointestinal symptom improvement, gluten degradation after a study meal; 2°: Health-related quality of life, gluten degradation after a study meal, clinically significant change in vital signs and physical examination	NCT04243551 Phase 2
***Synthetic Sequestrant***		
BL-7010	1°: Safety and tolerability; 2°: Plasma levels of BL-7010	NCT01990885 ^1^ Phase 1/2
***Anti-Gliadin Antibody Supplement***		
AGY	1°: Safety and tolerability; 2°: Gastrointestinal symptoms	NCT01765647 ^1^ Phase 1
	Changes in celiac-related symptoms	NCT03707730 Phase 2

^1^ Trial completed; ^2^ trial terminated; ^3^ trial suspended. Identifiers without status designation are in various stages of recruitment.

**Table 2:** Effect of polyphenols on gliadin-mediated inflammation and damage in vitro using Caco-2 cells as a model for celiac disease.

Protective Effects	Gliadin Source	Polyphenol Classification	Reference
Improved barrier integrity; reduced secretion of IL-6, IL-8	Pepsin-trypsin-digested gliadin	Green tea extract (653 mg g^−1^ catechins inc. 413 mg g^−1^ EGCG)	Van Buiten, Lambert and Elias (2018)
Reduced paracellular transport of gliadins	p58–89	EGCG	Dias, Brás, Fernandes, Pérez-Gregorio, Mateus and Freitas (2018)
		Procyanidin B3, procyanidin C2	Dias, Brás, Pérez-Gregorio, Fernandes, Mateus and Freitas (2019)
Reduced TG2 and COX-2 expression, reduced secretion of IL-15, IL-1β, IL-6, IL-8	p31–43	Cocoa extract (21.39 mg g^−1^ procyanidin B2)	Kramer, Yeboah-Awudzi, Magazine, King and Xu (2020)

## Data Availability

No new data were created or analyzed in this study. Data sharing is not applicable to this article.

## Abbreviations

IBD	Inflammatory bowel disease
NMR	Nuclear magnetic resonance
EGCG	Epigallocatechin gallate
PPII	Polyproline II helix
TG2	Tissue transglutaminase
IL	Interleukin
Ig	Immunoglobulin
